# Metamorphosis and development of malaria parasites in the liver are regulated by unconventional autophagy

**DOI:** 10.1080/27694127.2025.2504060

**Published:** 2025-05-11

**Authors:** Suryansh Rajput, Satish Mishra

**Affiliations:** a Division of Molecular Microbiology and Immunology, CSIR-Central Drug Research Institute, Lucknow, India; b Academy of Scientific and Innovative Research (AcSIR), Ghaziabad, India

**Keywords:** Apicoplast, autophagy, Atg7, Atg8, lipidation, liver stage, malaria, *Plasmodium*, sporozoites

## Abstract

Malaria parasites encounter diverse conditions as they transition between mosquito and mammalian hosts. A characteristic of the sporozoite stage of the parasite is that once it enters the hepatocyte, it changes its morphology and metabolism. Motile-elongated sporozoites transform into round trophozoites, discard unnecessary organelles, undergo extensive replication, and mature into hepatic merozoites. However, the mechanisms of superfluous organelle elimination and apicoplast biogenesis are unclear. In our latest study, using a conditional mutagenesis system, we clarified the role of Atg7 during parasite metamorphosis in the liver. We found that cytosolic Atg7 is essential for the localization of Atg8 on the membrane and the development of parasites in the blood and liver stages. Parasites lacking Atg7 fail to lipidate Atg8 on the membrane, which leads to impaired exocytosis of micronemes, and parasites eventually fail to mature into hepatic merozoites. This work provides insights into the essential role of Atg7 in maintaining parasite cellular homeostasis during liver stage development.

To maintain cellular homeostasis, eukaryotic cells possess a fundamental mechanism to self-eat and self-feed themselves during stress conditions called autophagy. This degradation and recycling phenomenon involves the sequestration of cytoplasmic components into double-membrane vesicles called autophagosomes, which ultimately fuse with the lysosome to degrade the cargo. The traditional role of autophagy in eukaryotes is well characterized in the context of nutrient stress and starvation. Partial conservation of the ATG genes in malaria parasites has modified the conventional autophagy pathway. The unconventional autophagy plays a significant role in shaping the survival and adaptation of the malaria parasite, which transitions between mosquito and mammalian hosts. This enables parasites to undergo stage-specific differentiation during their complex life cycle, which is characterized by metabolic reprogramming, intracellular remodeling, organellar biogenesis, and protein degradation. Following hepatocyte invasion, sporozoites are remarkably remodeled into round trophozoite stages, and the organelles that facilitate parasite invasion become unnecessary. The elimination of superfluous organelles was associated with the autophagy-related (ATG) protein Atg8. Despite having a complex life cycle, *Plasmodium* possesses fewer *ATG* genes than do higher eukaryotes. However, it preserves the well-documented tri-enzyme cascade, like ubiquitin‒proteasome pathway, which mediates the lipidation of Atg8. Atg8 and Atg12 are two ubiquitin-like proteins that canonically function in cargo recruitment and autophagosome formation but also play some noncanonical roles. The link between parasite autophagy processes and the unique organelle apicoplast is a fascinating area of study in the atg8 conjugation system.

The Atg12 and Atg8 conjugation systems can be dissected into a series of four main enzymatic reactions, namely, processing, activation, transfer, and conjugation. Newly synthesized Atg8 is post post-translationally processed by the cysteine protease Atg4 to cleave its C-terminal extension and expose a glycine residue essential for its activation. In contrast, Atg12 is already synthesized with an exposed glycine at its C-terminal tail. The exposed glycine residues of Atg8 and Atg12 are covalently attached via a thioester bond to the catalytic cysteine of the E1-like activating enzyme Atg7. In mammals, activated ATG12 is transferred to ATG10, but there is no evidence that the same occurs in *Plasmodium*. Owing to the existence and essentiality of the Atg12 protein in the parasite, it may be hypothesized that Atg7 alone or with an unknown interacting partner may facilitate the transfer of Atg12 to Atg5 and thus favor the assembly the parasite’s Atg12–Atg5-Atg16 E3 ligase complex. Activated Atg8 is transferred to the E2 conjugating enzyme Atg3, which together with the Atg12–Atg5-Atg16 E3 ligase complex conjugate Atg8 to the phosphatidylethanolamine present in the target membranes. During autophagy, lipidated Atg8 on the forming phagophore has various functions including acting as an adapter for cargo selection. The conjugation of Atg8 to the apicoplast membrane is essential for its biogenesis in malaria parasites.

To investigate the role of Atg8 and Atg12 conjugation systems in the *Plasmodium* life cycle, we attempted to delete Atg7 via conventional double crossover homologous recombination but failed to recover the mutant parasite [1]. Next, we proceeded with conditional silencing of the gene in the mosquito stage using the flippase (FLP)/flippase recognition target (FRT)-based conditional mutagenesis system. Mutant Atg7 sporozoites normally travel to mosquito salivary glands. However, Atg7-mutant sporozoites failed to initiate blood-stage infection in mice [1]. Next, we infected HepG2 cultures with Atg7-mutant sporozoites and demonstrated via immunofluorescence assays that the mutant parasites were arrested during liver stage development. Atg7 mutants not only presented significant growth defects but were also unable to form merozoites. Moreover, Atg7 mutants were unable to segregate their nuclei into individual merozoites. The average exoerythrocytic form (EEF) size, area, and nuclear count were significantly lower in the Atg7 mutant parasites than in the control parasites [1]. Parasites lacking Atg7 fail to discard superfluous organelles and mature into hepatic merozoites. The apicoplast, the relict plastid, which is thought to branch via Atg8 lipidation, was unable to form an extensive network in the absence of Atg8 conjugation. Atg7 mutant parasites immunostained with the apicoplast marker ACP (acyl carrier protein) presented the remnants of the organelle that persisted in the knockout but failed to branch extensively due to a lack of Atg8 conjugation. Similarly, the formation and branching of the endoplasmic reticulum were also affected in Atg7 knockout parasites [1]. We did not investigate the fate of mitochondria in the Atg7 knockout but owing to the pleiotropic nature of Atg7 and the above-described findings, we speculate that mitochondrial biogenesis may also be affected. Next, we monitored the distribution and fate of micronemes from sporozoites to the late liver-stage development of the parasite. This was revealed by immunostaining sporozoites and EEFs with TRAP (thrombospondin-related anonymous protein), a marker for parasite micronemal proteins. Our data revealed that after sporozoite invasion into the liver stage, micronemes fail to exocytose in Atg7 mutant parasites (Figure 1).

**Figure 1.**
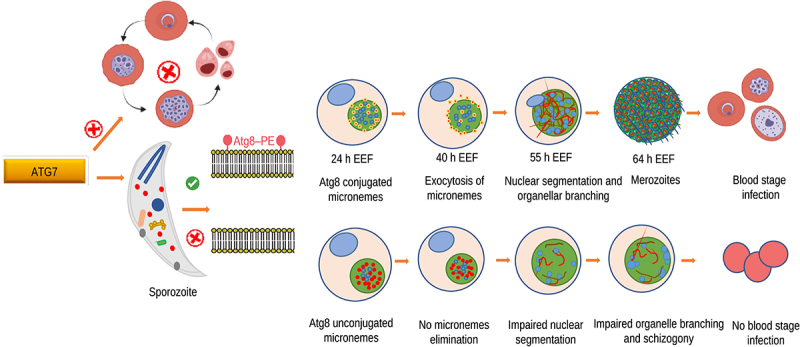
Proposed model illustrating how Atg7 regulates parasite development.

Having established the essential and multifunctional roles of Atg7 in *P. berghei*, the ATG proteins in *Plasmodium* offer significant opportunities to explore new drug targets. Furthermore, we are in the process of identifying potential inhibitors against Atg7 to kill blood- and liver-stage parasites. A successful Atg7 inhibitor should block microneme clearance and parasite maturation. Our future work will address the development of target-specific inhibitors to prepare multistage drugs to alleviate malaria. Future studies exploring the role of other ATG proteins involved in Atg8 conjugation and deconjugation in malaria parasites may reveal new therapeutic opportunities.

## Abbreviations

ATG: Autophagy-related
EEF: exoerythrocytic form
ACP: acyl carrier protein
FLP: flippase
FRT: flippase recognition target
TRAP: thrombospondin-related anonymous protein.
